# Treating Seizures after Hypoxic-Ischemic Encephalopathy—Current Controversies and Future Directions

**DOI:** 10.3390/ijms22137121

**Published:** 2021-07-01

**Authors:** Kelly Q. Zhou, Alice McDouall, Paul P. Drury, Christopher A. Lear, Kenta H. T. Cho, Laura Bennet, Alistair J. Gunn, Joanne O. Davidson

**Affiliations:** The Department of Physiology, The University of Auckland, Auckland 1023, New Zealand; k.zhou@auckland.ac.nz (K.Q.Z.); a.mcdouall@auckland.ac.nz (A.M.); p.drury@auckland.ac.nz (P.P.D.); christopher.lear@auckland.ac.nz (C.A.L.); kenta.cho@auckland.ac.nz (K.H.T.C.); l.bennet@auckland.ac.nz (L.B.); aj.gunn@auckland.ac.nz (A.J.G.)

**Keywords:** hypoxic-ischemic encephalopathy, asphyxia, seizures, antiepileptic drugs, anticonvulsants, therapeutic hypothermia, phenobarbital, levetiracetam

## Abstract

Seizures are common in newborn infants with hypoxic-ischemic encephalopathy and are highly associated with adverse neurodevelopmental outcomes. The impact of seizure activity on the developing brain and the most effective way to manage these seizures remain surprisingly poorly understood, particularly in the era of therapeutic hypothermia. Critically, the extent to which seizures exacerbate brain injury or merely reflect the underlying evolution of injury is unclear. Current anticonvulsants, such as phenobarbital and phenytoin have poor efficacy and preclinical studies suggest that most anticonvulsants are associated with adverse effects on the developing brain. Levetiracetam seems to have less potential neurotoxic effects than other anticonvulsants but may not be more effective. Given that therapeutic hypothermia itself has significant anticonvulsant effects, randomized controlled trials of anticonvulsants combined with therapeutic hypothermia, are required to properly determine the safety and efficacy of these drugs. Small clinical studies suggest that prophylactic phenobarbital administration may improve neurodevelopmental outcomes compared to delayed administration; however, larger high-quality studies are required to confirm this. In conclusion, there is a distinct lack of high-quality evidence for whether and to what extent neonatal seizures exacerbate brain damage after hypoxia-ischemia and how best to manage them in the era of therapeutic hypothermia.

## 1. Neonatal Seizures after Hypoxic-Ischemic Encephalopathy

Loss of oxygen (hypoxia) and blood supply (ischemia) to the brain can occur before, during or shortly after birth. Moderate to severe hypoxic-ischemic encephalopathy (HIE) occurs in ~1 to 3/1000 live births in developed nations and can lead to death, or in survivors, brain damage with lifelong disability, including cerebral palsy and epilepsy [1]. Induced mild “therapeutic” hypothermia (cooling) is now established to significantly improve survival without disability, but many infants still survive with disability despite treatment [2]. There are many unanswered questions about how to manage infants during therapeutic hypothermia, particularly whether add-on anticonvulsant therapy is necessary or beneficial [3].

Seizures are common in newborn infants with HIE. For example, in a study of electroencephalographic (EEG) monitoring in 47 neonates with HIE, 62% had seizures [4]. Seizure burden is consistently associated with both brain injury and adverse neurodevelopmental outcome [5,6]. However, the direction of causality is unclear, and the consequences of seizure activity for the developing brain and the most effective way to manage these seizures remain surprisingly poorly understood. Although seizures in the neonate are largely regarded as a neurologic emergency requiring prompt management, in practice there is little evidence supporting a beneficial effect of anticonvulsants and there is concerning preclinical evidence that many anticonvulsants may have neurotoxic effects on the developing brain [7]. Furthermore, it is not clear how the relative benefits and risks of anticonvulsants have changed in the era of therapeutic hypothermia [7]. Thus, optimal management of seizures remains controversial.

## 2. The Biology of Neonatal Seizures

The mechanisms of seizures in the neonate appear to be different in many ways to those in the adult or even during childhood. The majority of neonatal seizures occur as a transient response to acute cerebral pathology and are most commonly associated with HIE (38%), ischemic stroke (18%) and intracranial hemorrhage (11%) [8]. In infants with HIE, seizures usually start hours after birth, their intensity reflects the severity of underlying cerebral injury and they tend to resolve over hours to days irrespective of seizure management [4].

Biologically, there are differences in neurotransmitter function during development that may increase the susceptibility of the immature brain to seizures compared to adults and have the potential to affect the efficacy of anticonvulsants. Early in development, gamma-aminobutyric acid (GABA) receptors may respond to stimulation with excitation, rather than the inhibition observed in the adult [9]. This difference is due to a high intracellular Cl^-^ concentration resulting from high Na-K-2Cl (NKCC1) expression, which mediates Cl^-^ entry and low K-Cl cotransporter KCC2 expression, which mediates Cl^-^ exit from cells [10,11]. Following the upregulation of the potassium-chloride co-transporter KCC2 during the early postnatal period, Cl^-^ can be extruded from cells and therefore GABA and glycine become inhibitory. This relative increase in excitability is important for the development of neural circuitry, but likely increases susceptibility to seizures [12]. Further, there is evidence in juvenile rats that hypoxia-ischemia late in gestation reduced neuronal KCC2 expression, impairing hippocampal CA3 inhibitory tone [13]. Impaired GABAergic signaling after hypoxia-ischemia may lower the seizure threshold, contributing to reduced efficacy of GABAergic agonists such as phenobarbital in neonates.

Moreover, the immature brain has a greater density of calcium permeable, GluR2-subunit-deficient AMPA receptors, which contribute to a lower threshold for seizures, and expression of NMDA glutamate receptor subunits (GluN) such as GluN2B, which promote prolonged excitatory post-synaptic potentials and GluN3A [14]. Further, hypoxia-ischemia can change the expression of NMDA receptor subunits, therefore promoting seizures in the developing brain [15].

These biological differences in the neonatal brain compared to the adult are still being explored, but they may have consequences for the initiation and propagation of seizure activity as well as the efficacy and long-term neurodevelopmental effects of anticonvulsants. Thus, it is vital to better understand the pathophysiology of neonatal seizures and develop and test anticonvulsant protocols specifically in neonates, and not rely on evidence from studies in adults.

## 3. Do Seizures Exacerbate Brain Damage after HIE?

Understanding whether seizures independently contribute to brain damage after HIE, or merely reflect the underlying evolution of injury resulting from a period of hypoxia-ischemia is crucial for determining how aggressively seizures should be managed. The effects of seizure activity on the severity of brain damage after HIE are complex and poorly understood, with much conflicting evidence. Much of this uncertainty stems from the inherent difficulty in trying to detangle the direct effects of seizures on brain injury from indirect effects mediated by the severity of the underlying hypoxic-ischemic injury that triggered the seizures in the first place.

There is some, limited clinical evidence that seizures may exacerbate injury in infants with HIE. A clinical magnetic resonance spectroscopy study suggested that seizures were associated with a mismatch in oxygen supply and demand, such that increased seizure burden was associated with elevated lactate and reduced NAA/choline ratio, a marker of neuronal injury [16]. This seems to indicate that the severity of seizures in human newborns with HIE is independently associated with brain injury [16]. Consistent with this hypothesis, a small prospective study found that infants with clinical seizures had worse motor and cognitive outcomes after controlling for injury severity seen on magnetic resonance imaging (MRI) 5 days after birth [17], and further, that greater seizure severity was associated with worse motor and cognitive outcomes. More recently, two small, randomized trials of management of clinical vs. EEG-proven seizures have suggested that higher seizure burden is associated with greater brain injury and worse neurodevelopmental outcomes [18,19]. Although these data seem to suggest that higher seizure burden may be harmful, the effect of greater management during EEG monitoring in these studies on seizures was not statistically significant. Recently, a study of predictive models for death and neurodevelopmental impairment for infants with HIE, found that seizures were not independently predictive of outcome, due to collinearity with injury severity [20]. More generally, the clinical impact of a mild to moderate seizure burden is still unclear and the threshold that should prompt management in routine clinical care is unknown.

A limitation of these studies is that imaging reflects a single time point, while seizures reflect a dynamic, evolving process. Critically, these studies are not able to determine the direction of causality. That is to say, it is possible that more severe underlying injury could have reduced NAA expression [16], and increased both seizure burden and risk of adverse neurodevelopmental outcome. Supporting this hypothesis, a secondary analysis of the National Institute of Child Health and Human Development (NICHD) whole body hypothermia trial found that after adjusting for treatment group and the severity of HIE, there was no significant association between clinical seizures and death, or moderate or severe disability or lower Bayley Mental Development Index score at 18 months of life [21]. Acknowledging that this was a post hoc analysis, these data suggest the hypothesis that among infants with HIE, the mortality and morbidity often attributed to seizures could be better explained by the underlying severity of encephalopathy.

There has also been speculation that neonatal seizures prime the brain for later development of epilepsy. In rodents, neonatal seizures are associated with long-lasting changes including reduced neurogenesis, sprouting of mossy fibers, and altered cell firing, as previously reviewed [22]. Clinically, in a study of 92 term infants with HIE, there was a significant association between neonatal seizures and subsequent development of epilepsy [23]. However, this association was no longer significant after adjusting for the severity of HIE, such that only the presence of severe HIE was independently associated with the later development of epilepsy [23]. Supporting this hypothesis, there is evidence from a cohort study of reduced rates of epilepsy in infancy and childhood after treatment of HIE with therapeutic hypothermia [24].

## 4. The Physiology of Seizures

The most likely way that seizures could exacerbate brain injury is by excessively increasing local brain metabolism leading to tissue hypoxia and other metabolic deficits. In normal, well-oxygenated adult rats, gas flurothyl-induced status epilepticus was associated with localized neuronal necrosis after as little as 30 min (Table 1) [25]. Subsequent studies have shown that the severity of neuronal injury after induced status epilepticus is highly affected by brain maturity, and the extent of injury is rather variable between models. Although some models of induced neonatal seizures are associated with significant damage [26], others are associated with rather mild injury [27], with subtle or even no long-term effects on social behavior or cognition [28,29]. Interestingly, exposure to infection/inflammation may be an important factor that greatly increases seizure induced damage in postnatal day (P)7 and P14 rat pups [27].

Some of this heterogeneity in experimental studies may reflect the specific pattern of seizures. There is evidence, for example, that large amplitude but discrete seizures may not significantly compromise tissue oxygenation, emphasizing that not all seizures are the same. In near-term fetal sheep exposed to asphyxia induced by 10 min of complete umbilical cord occlusion, seizures that lasted less than 3.5 min in duration were not associated with altered tissue PO_2_, as measured by laser Doppler on the parietal cortex [30]. However, seizures lasting longer than 3.5 min were associated with a small but significant fall in tissue PO_2_, which plateaued in association with a delayed increase in carotid artery blood flow. Although these findings suggest a mismatch between oxygen supply and demand occurs during seizures longer than 3.5 min, this mismatch was relatively mild, with no further exacerbation with increasing seizure length [30].

If seizures did cause brain injury after hypoxia-ischemia, then it follows that preventing seizures should prevent the development of injury. However, blockade of the NMDA receptor using the highly selective non-competitive glutamate receptor antagonist, dizocilpine, between 6 and 24 h after global cerebral ischemia in the near-term fetal sheep suppressed brain activity and completely prevented post-ischemic seizure activity. Despite this, it was not associated with any improvement in the neuronal loss in the parietal cortex, the most severely affected region in this paradigm [31]. There was a small reduction in neuronal loss in less severely affected regions, such as the hippocampus and lateral cortex, suggesting that seizures may play a role in exacerbating cell death in regions outside of the primary injury, although it is possible that the effect was due to the direct anti-excitotoxic effect of dizocilpine. Similarly, in P7 rats, neonatal stroke was associated with both high amplitude EEG spikes during ischemia and stereotypic seizures after reperfusion [32]. Administration of either phenobarbital or levetiracetam suppressed the high amplitude spikes and phenobarbital delayed the onset of seizures, but neither reduced the volume of infarction. These data strongly suggest that preventing or at least suppressing seizures does not prevent bulk neuronal cell death after hypoxia-ischemia but may reduce cell death in mildly affected regions, at least in the normothermic brain.

Consistent with this, in neonatal piglets exposed to moderate to severe hypoxia-ischemia, although energy metabolism was disturbed in both grey and white matter, likely due to mitochondrial dysfunction secondary to evolving hypoxic-ischemic brain injury, seizure activity did not appear to exacerbate injury [33]. Further, in P7 rats, increasing seizure burden after neonatal stroke with a lithium-pilocarpine-induced status epilepticus did not increase infarct volume [32]. Further, in normal P10 rats, seizures induced by injection of the excitotoxin kainic acid induced clinical and electrographic seizures lasting a mean of 282 min, but did not cause brain injury after either 3 or 20 days recovery [34]. In this study, mild hypoxia-ischemia for 30 min was associated with moderate neuropathological injury but no electrographic seizures. Kainic acid injection after hypoxia-ischemia induced superimposed seizures and mildly increased neuronal loss in the hippocampus. Critically, however, these induced seizures were associated with a small increase in brain temperature—and preventing this hyperthermia abolished the increase in neuronal necrosis up to 20 days after hypoxia-ischemia [35]. Thus, induced seizures per se may not significantly increase brain injury after hypoxia-ischemia and a significant part of any injurious effect may be mediated by hyperthermia, consistent with the association of pyrexia with greater brain injury and adverse neurodevelopmental outcomes in multiple preclinical studies and clinical trials of babies with neonatal encephalopathy [36,37]. These data strongly suggest that seizures were largely bystanders to the overall evolving injury rather than a major contributing factor.

**Table 1:** Summary of preclinical studies.

Study Aim	Animal Species and Age	Study Outcomes	Reference
Seizures and brain injury	Adult rat	Gas flurothyl-induced status epilepticus was associated with neuronal necrosis.	[25]
	P7 rat	High-dose lithium and pilocarpine induced status epilepticus was associated with widespread brain injury.	[26]
	P7 and P14 rat	High-dose lithium and pilocarpine induced status epilepticus was associated with selective hippocampal damage which was exacerbated with lipopolysaccharide pretreatment.	[27]
	P9	Pilocarpine-induced status epilepticus at P9 was associated with impaired social behavior at P60.	[28]
	Neonatal rat	Single episode of pilocarpine-induced status epilepticus at neonatal age was not associated with impaired cognitive function assessed at P60 to P63.	[29]
	Near-term fetal sheep	Post-asphyxial seizures lasting longer than 3.5 min resulted in a drop in tissue PO_2_, but there was no further exacerbation with longer seizures.	[30]
	Near-term fetal sheep	NMDA receptor blockade with dizocilpine at 6–24 h after global cerebral ischemia prevented seizures, improved neuronal survival in the lateral cortex and hippocampus but not the parietal cortex (most injured area).	[31]
	P7 rat	Phenobarbital or levetiracetam suppressed high amplitude spikes after neonatal stroke, but this did not reduce brain infarction volume.	[32]
	Neonatal piglet	Seizure activity did not increase cerebral lactate or lactate/pyruvate ratio above the increased levels seen after hypoxia-ischemia, therefore unlikely to exacerbate injury.	[33]
	P10 rat	Kainic acid-induced seizures alone were not associated with brain injury, but the combination of hypoxia-ischemia and kainic acid increased hippocampal injury.	[34]
	P10 rat	Preventing hyperthermia during seizures induced by hypoxia-ischemia plus kainic acid, reduced brain injury compared spontaneously hyperthermic animals.	[35]
Phenobarbital efficacy	P11	Phenobarbital suppressed seizures when administered before, but not after hypoxia-ischemia.	[38]
Phenobarbital neuroprotection	P7 rat	Early administration of phenobarbital with hypothermia after hypoxia-ischemia was associated with better sensorimotor performance, lower neuropathology scores and reduced infarct volume compared to hypothermia alone.	[39]
	P10 rat	Early administration of phenobarbital with hypothermia after hypoxia-ischemia was associated with improved motor outcome and brain injury.	[40]
Anticonvulsant adverse effects	Neonatal macaques	Phenobarbital infusion followed by midazolam administration was associated with widespread apoptosis, which was exacerbated with longer exposure. Further injury continued to evolve over time.	[41]
	P4 rat	Phenobarbital administration was associated with reduced proliferation, reduced expression of neuronal markers and transcription factors, and neurotrophins.	[42]
	P0-P30 rat	Administration of phenytoin, phenobarbital, diazepam, clonazepam, vigabatrin or valproate all independently induced widespread neuronal apoptosis, which was dose-dependent.	[43]
	P7 or P10 rat	Administration of either phenobarbital, phenytoin or lamotrigine but not levetiracetam was associated with impaired striatal synaptic development between P10 and P18.	[44]
	P7 rat	Levetiracetam administration did not induce cell death in the brain.	[45]

## 5. Anticonvulsants for the Management of Neonatal Seizures

A further consideration is that even if in principle it was desirable to use anticonvulsants, currently available therapies are notably ineffective (Table 2). A widely recommended first-line drug for neonatal seizures is the barbiturate, phenobarbital, which acts by increasing chloride currents through GABA receptors, increasing neuroinhibition [46]. If this does not provide effective seizure control, second-line agents such as phenytoin and levetiracetam, and third-line interventions (e.g., midazolam infusions) may be needed [46]. However, these anticonvulsants have poor efficacy, likely in part due to the differences between the pathophysiology of adult and neonatal seizures. In a randomized controlled trial of 59 neonates with seizures, phenobarbital or phenytoin were effective in controlling seizures in less than half the infants [47]. Consistent with this, in a recent prospective study of 611 preterm and term neonates presenting with seizures, 63% failed to respond to an initial loading dose of phenobarbital [48]. The neonates that were most likely to respond to phenobarbital were those with mild seizures or seizures that were already decreasing in severity before administration. The evidence for a beneficial effect of anti-seizure drugs in neonates is so limited that a 2007 Cochrane review concluded that *“anticonvulsant therapy to term infants in the immediate period following perinatal asphyxia cannot be recommended for routine clinical practice, other than in the treatment of prolonged or frequent clinical seizures*” [49]. Similarly, a recent systematic review has confirmed that there is still a distinct lack of evidence to guide the use of anticonvulsants for neonatal seizures [50]. Despite this lack of evidence, aggressive treatment of neonatal seizures remains routine clinical practice.

The desire to find more effective and safer neonatal anticonvulsants has supported studies of newer anticonvulsants. A leading candidate is the novel anticonvulsant, levetiracetam, either as a potential add-on treatment or as first-line treatment for neonatal seizures. Levetiracetam works by modulating synaptic neurotransmitter release through binding to the synaptic vesicle (SV) protein SV2A, with greatest effect in rapidly discharging neurons [51]. However, the evidence for the efficacy of levetiracetam as a first-line treatment for reducing neonatal seizures is mixed, with some studies suggesting that it is not any more effective, and may in fact be substantially worse, than routinely used first-line agents such as phenobarbital. 

A small retrospective study of 23 neonates with seizures who received levetiracetam showed a greater than 50% seizure reduction in only 35% (8/23) of patients in the cohort [52]. Similarly, levetiracetam monotherapy provided seizure control in 47% of patients in a small retrospective study of 36 neonates [53]. By contrast, a small one-blind prospective study in term neonates with seizures, randomized to phenobarbital or levetiracetam, showed a significant improvement in tone and posture at one month follow-up in the levetiracetam group but no information was presented on the efficacy of each drug to reduce seizure activity [54]. In a recent open label randomized controlled trial of 100 neonates presenting with clinical seizures, seizures stopped in 86% of those randomized to levetiracetam and 62% to phenobarbital (*p* < 0.01) [55]. A recent systematic review of levetiracetam as a first-line treatment of neonatal seizures concluded that levetiracetam and phenobarbital were equally effective for seizure control. However, overall, levetiracetam seemed to be associated with a lower risk of adverse events [56].

In a recent phase IIb randomized controlled trial (NEOLEV trial), neonates received either phenobarbital or levetiracetam as first-line treatment for neonatal seizures from any cause [57]. 80% of patients who received phenobarbital, but only 28% of patients who received levetiracetam, remained seizure free for 24 h. Sub-analysis of HIE patients treated with hypothermia showed that 90% of those who received phenobarbital but only 35% of those who received levetiracetam had 24 h seizure cessation. Interestingly, the efficacy of phenobarbital was very high in this study, likely relating to their use of continuous EEG monitoring and a real-time response to seizure detection [58]. This suggests that early administration of phenobarbital may be more effective than later administration.

**Table 2:** Summary of clinical studies of anticonvulsant therapy.

Study Aim	Study Type	Study Outcome	Number of Participants	Reference
Seizures and outcome	Observational	High seizure burden in babies with HIE were associated with abnormal outcome, with or without hypothermia.	47	[4]
	Observational	HIE infants treated with hypothermia with clinical seizures had more extensive injury on MRI scans and delayed neurodevelopment at 18–24 months.	97	[5]
	Observational	High seizure burden and persistent abnormal aEEG background in HIE infants treated with hypothermia was associated with poor prognosis.	30	[6]
	Observational	High seizure burden was associated with higher mortality and abnormal neurological exam at discharge in infants with HIE, ischemic stroke or intracranial hemorrhage.	426	[8]
	Observational	Seizure severity in newborns with perinatal asphyxia was independently associated with brain injury.	90	[16]
	Observational	Clinical seizures were are associated with worse neurodevelopmental outcome, independent of hypoxic-ischemic injury severity.	77	[17]
	Observational	Clinical seizures were not associated with death, disability or lower developmental scores after adjusting for HIE severity.	208	[21]
	Observational	Seizures were not independently predictive of outcome, due to collinearity with HIE severity.	486	[20]
Treating electrographic and clinical seizures, or clinical seizures only	RCT	EEG monitoring for treatment of electrographic seizures in HIE infants was associated with a reduction in seizure burden. Higher seizure burden is associated with more severe brain injury and lower neurodevelopment scores at 18 to 24 months.	69	[18]
	RCT	Trend for reduction in seizure duration when treating electrographic seizures. Seizure duration is associated with severity of brain injury.	42	[19]
HIE and epilepsy	Observational	Infants with severe but not moderate HIE were associated with developing epilepsy at 24 months.	92	[23]
Hypothermia treatment and epilepsy	Observational	Reduced rates of epilepsy up to 8 years of age in cohort treated with hypothermia for HIE.	151	[24]
Phenobarbital efficacy	Observational	Subclinical seizures were more common in preterm infants. 63% of preterm and term infants with seizures failed to respond to phenobarbital.	611	[48]
	RCT	Phenobarbital was associated with a 27% reduction in incidence of seizures for neonates with severe asphyxia.	31	[59]
Phenobarbital vs. phenytoin efficacy	RCT	Either phenobarbital or phenytoin controlled seizures in less than half of the neonates.	59	[47]
Effectiveness of levetiracetam	Observational	Levetiracetam was associated with reducing 50% of seizures in 35% of infants.	23	[52]
	Observational	Levetiracetam monotherapy provided seizure control in 47% of infants.	36	[53]
Phenobarbital vs. levetiracetam efficacy	RCT	Improvement in tone and posture of infants treated with levetiracetam but not phenobarbital.	30	[54]
	RCT	First-line levetiracetam achieved better seizure control than phenobarbital for neonatal seizures.	100	[55]
	RCT	First-line phenobarbital treatment was more effective than levetiracetam for neonatal seizures.	85	[57]
Hypothermia efficacy for seizures	Observational	Hypothermia reduced seizure burden for neonates with moderate HIE.	107	[60]
	Observational	Hypothermia reduced seizures for infants with HIE at 6 months follow up.	56	[61]
	Observational	0/5 neonates with stroke treated with hypothermia had seizures, compared to 7/10 who were not treated with hypothermia.	15	[62]
	Observational	Neonates born in a tertiary cooling center had fewer seizures and improved seizure-free survival compared to those born in a non-cooling center without active therapeutic hypothermia.	5059	[63]
Bumetanide efficacy	RCT	Bumetanide add-on to phenobarbital for treatment of neonatal seizures did not improve seizure control and increased the risk of hearing loss.	30	[64]
		Phenobarbital plus bumetanide for treatment of seizures in neonates with HIE showed reduced seizure burden compared to phenobarbital plus placebo.	53	[65]
Anticonvulsant adverse effects	Observational	Phenobarbital for the treatment of febrile seizures is associated with lower language/verbal scores at school age, and did not reduce the rate of seizure reoccurrences.	139	[66]
	Observational	Anticonvulsant use for infants with moderate/severe HIE were independently associated with death/disability at 18 months.	208	[67]

## 6. Should We Try to Prevent Seizures?

There is a lack of evidence that phenobarbital or any other anticonvulsant improves outcomes after neonatal seizures have already started. However, there is some clinical and preclinical evidence that prophylactic phenobarbital administration, that is to say administered before the appearance of seizure activity, may be beneficial [68]. Any benefit could be mediated by improved seizure control as phenobarbital suppressed seizures when administered before, but not after hypoxia-ischemia in P11 rats (Table 1) [38]. Although brain injury was not assessed in this study [38], there is intriguing evidence that phenobarbital may have neuroprotective effects independent of its anticonvulsant properties. For example, a small prospective study, conducted in the pre-hypothermia era, found that prophylactic high-dose phenobarbital administration (40 mg/kg) was associated with improved neurological outcome at three years of age, despite only showing a 27% reduction in seizures compared to neonates who received phenobarbital after seizures had started [59]. Further supporting the efficacy of prophylactic phenobarbital, a recent Cochrane review of prophylactic barbiturates (8/9 studies used phenobarbital) for infants with perinatal HIE [68], found that prophylactic barbiturate therapy reduced the risk of seizures after hypoxia-ischemia, with no reduction in mortality. However, there was little data on long-term outcomes. They concluded that *“the results of the current review support the use of prophylactic barbiturate therapy as a promising area of research”* although they could not recommend routine clinical use at this stage due to the low quality of evidence.

**Figure 1:**
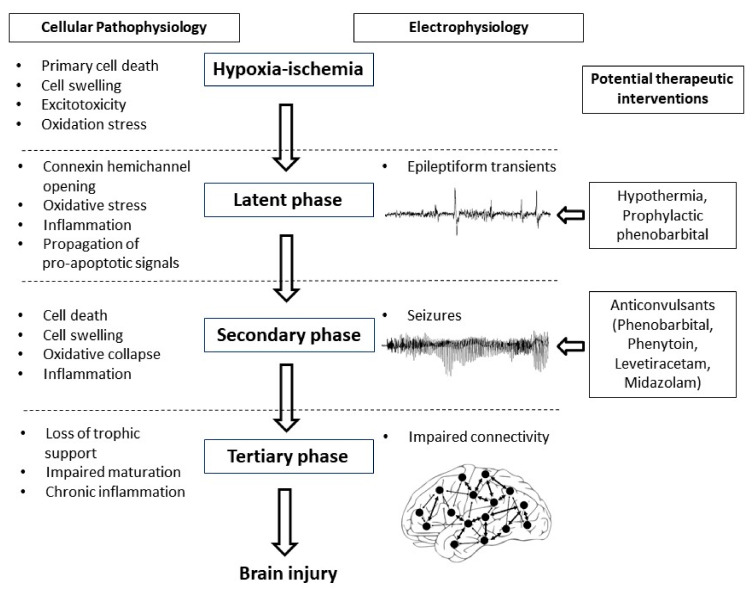
Flow diagram showing the phases of injury including hypoxia-ischemia, the latent phase, the secondary phase and the tertiary phase leading to the development of brain injury. Intervention during the latent phase with therapeutic hypothermia or prophylactic phenobarbital has the potential to reduce the development of brain injury as well as the occurrence of seizures. Intervention during the secondary phase with anticonvulsants such as phenobarbital, phenytoin, levetiracetam and midazolam may reduce seizure activity but their effects on long-term outcome are not clear.

The potential for early but not later administration of phenobarbital to be neuroprotective, likely relates to the well-characterized progressive evolution of hypoxic-ischemic brain injury (Figure 1) [3]. During the period of hypoxia-ischemia itself, there is often only limited cell death. Many brain cells partially or even completely recover after reperfusion, in a latent phase that lasts approximately 6 h after hypoxia-ischemia, only to undergo secondary deterioration as shown by delayed onset of seizures, cell swelling and bulk cell death from 6–72 h after hypoxia-ischemia [3]. It is now well established that therapeutic hypothermia needs to be started during this latent phase, before the onset of seizures (and secondary cell death), in order to be effective [69]. This suggests that when phenobarbital is administered after the onset of seizures (i.e., during the secondary phase), the window of opportunity for neuroprotection has already closed and so phenobarbital can only act as an anticonvulsant. It is plausible but unproven that administration of prophylactic phenobarbital during the latent phase, corresponding with the established window of opportunity for therapeutic hypothermia [69], may offer direct neuroprotection, and so reduce secondary cell death, leading to fewer seizures.

There is some evidence to suggest that early phenobarbital can augment the beneficial effects of therapeutic hypothermia. In P7 rats exposed to hypoxia-ischemia induced by single carotid artery ligation and inhalation hypoxia, phenobarbital augmented hypothermic neuroprotection [39]. Similarly, in P10 rat pups, Krishna et al. also found augmentation of hypothermic neuroprotection with prophylactic phenobarbital subjected to unilateral hypoxia-ischemia [40]. Although these studies are limited by the use of sub-optimal durations of hypothermia (3 to 4 h instead of the 72 h in established clinical protocols), they support the concept that prophylactic phenobarbital administration may have additive neuroprotective effects with therapeutic hypothermia. Strikingly, there no large animal, translational studies to date have tested whether there is benefit from combined treatment with prophylactic phenobarbital and hypothermia.

## 7. Therapeutic Hypothermia and Seizures

There is increasing clinical and preclinical evidence that therapeutic hypothermia per se reduces seizure activity in addition to its long-term neuroprotective effects. A study of 107 neonates with HIE showed that although hypothermia did not reduce the number of neonates with seizures, it was associated with a significant reduction in seizure burden in neonates with moderate, but not severe, HIE (Table 2) [60]. Further, a retrospective study of 56 neonates treated with therapeutic hypothermia for HIE showed that treatment with hypothermia was associated with a significant reduction in seizures within 6 months of discharge [61]. A recent retrospective cohort study of 5059 infants showed that those born in a tertiary cooling center had fewer seizures and improved seizure-free survival compared to those born in a non-cooling center without active therapeutic hypothermia [63]. Consistent with these findings, in a cohort study of 15 neonates with isolated focal ischemic stroke, 0/5 children treated with hypothermia had seizures compared to 7/10 who were not treated with hypothermia [62]. It is likely that the effect of hypothermia on seizures reflects both direct suppression of epileptiform activity combined with indirect effects mediated by stabilizing cell survival [70].

By contrast, a clinical pilot study of 14 neonates with HIE who were administered the loop diuretic bumetanide, which activates both renal and cerebral NKCC1, in conjunction with therapeutic hypothermia and phenobarbital was terminated early due to lack of prespecified efficacy and the finding that 3 infants developed hearing loss likely related to oto-toxicity, leading to an unacceptable risk to benefit ratio [64]. A more recent randomized controlled trial (*n* = 53) where neonates with HIE who have already received phenobarbital were given additional phenobarbital and bumetanide or placebo reported an additional reduction in seizure burden associated with bumetanide [65]. However, conclusive proof of efficacy is not yet available, and sufficiently powered phase 3 trials are needed. These studies highlighted a number of key challenges for future trials of anticonvulsants in the era of therapeutic hypothermia, including the fact that when treated with hypothermia, the number of neonates who developed seizures was low and likely included a high proportion of neonates with relatively severe brain injury, who were less likely to benefit from treatment with either therapeutic hypothermia or anticonvulsants.

Key challenges to improving management of seizures include the poor efficacy of current anticonvulsants for controlling neonatal seizures, the lack of evidence that they improve long-term outcome, concerns over adverse effects and uncertainty over their pharmacokinetics when administered in conjunction with therapeutic hypothermia. Given these issues, designing trials of new anticonvulsants in the hypothermia era using an “add-on” method, in combination with both hypothermia and phenobarbital is less than ideal and is likely to leave many important questions unanswered. Given that hypothermia is associated with reduced overall seizure burden, improves long-term outcomes and is safe for use in neonates with HIE in intensive care, we propose that therapeutic hypothermia alone should be considered to be the standard treatment rather than hypothermia plus phenobarbital, and that anticonvulsants should be randomized on top of hypothermia [7]. This would allow researchers to finally establish which anticonvulsant or combination of anticonvulsants is most effective for use with therapeutic hypothermia in infants with HIE. It is also important to include long-term follow-up in these studies, to determine whether the use of anticonvulsants on top of therapeutic hypothermia is associated with any beneficial or detrimental long-term neurological effects.

## 8. Are Anticonvulsants Toxic in the Developing Brain?

Animal studies suggest that anticonvulsants have the potential to cause unwanted side-effects in the developing brain, including widespread apoptotic neurodegeneration and altered neuronal proliferation. For example, in the neonatal macaque, administration of phenobarbital followed by infusion of midazolam over 5 or 24 h was associated with widespread apoptosis affecting both neurons and oligodendrocytes, with more extensive neurodegeneration after longer exposure [41]. Further, neuronal apoptosis continued to evolve over time, with areas of the brain not affected at 8 h showing increased neuronal apoptosis at 36 h with evidence of degeneration of neuronal tracts and trans-neuronal death of neurons, presumed to be resulting from their disconnection from degenerated pre-synaptic partners [41]. Consistent with this, phenobarbital administration in P4–P6 neonatal rats was associated with a significant reduction in proliferation in the dentate gyrus and reduced expression of neuronal markers, neuronal transcription factors and neurotrophins [42]. In P7 rats, administration of phenytoin, phenobarbital, diazepam, clonazepam, vigabatrin or valproate individually to achieve plasma concentrations consistent with those used clinically to control neonatal seizures was associated with widespread apoptotic degeneration of neurons in a dose-dependent manner, reduced expression of pro-survival neurotrophins, and a significant reduction in brain weight after eight days [43]. Further, clinically relevant doses of phenobarbital, phenytoin and lamotrigine individually in P7 neonatal rats were associated with impaired striatal synaptic development between P10 and P18 [44]. However, levetiracetam treatment was not found to impair synaptic development [44]. This is consistent with a previous study that showed that levetiracetam alone did not induce cell death in the P7 rat brain [45]. These encouraging studies suggest that levetiracetam may be associated with less toxic effects in the developing brain compared to other anticonvulsants.

Although it is difficult to determine whether anticonvulsants have acute neurotoxic effects in human neonates, long-term exposure to phenobarbital for the management of febrile seizures, was associated with an impairment in the development of language/verbal skills by school age with no difference in the recurrence of febrile seizures [66]. In a secondary analysis of the NICHD therapeutic hypothermia randomized controlled trial, severe HIE, anticonvulsants and mechanical ventilation were independently associated with death/disability at 18 month, whereas therapeutic hypothermia was protective [67].

## 9. Conclusions

Current anticonvulsant protocols are clearly far from optimal for use in neonates—they show limited efficacy, have high potential for adverse effects and there is a striking lack of evidence that they improve long-term outcomes. Preclinical neurophysiological studies are essential to help to better understand the pathophysiology of seizures occurring in the neonatal brain compared to the mature brain and identify specific anticonvulsant treatment strategies for the neonatal brain. High quality translational studies will help to answer questions around whether prophylactic anticonvulsant treatment is neuroprotective and more effective than delayed administration and whether anticonvulsants are associated with neurotoxic effects when given in combination with therapeutic hypothermia. Well-designed randomized controlled trials, taking into account the use of therapeutic hypothermia, are essential to develop evidence-based treatment strategies for the neonate with seizures.

## Data Availability

Not applicable.
